# Mechanotransduction Regulates Reprogramming Enhancement in Adherent 3D Keratocyte Cultures

**DOI:** 10.3389/fbioe.2021.709488

**Published:** 2021-09-10

**Authors:** Shenyang Li, Chengcheng Ding, Yonglong Guo, Yanan Zhang, Hao Wang, Xihao Sun, Jun Zhang, Zekai Cui, Jiansu Chen

**Affiliations:** ^1^ Aier School of Ophthalmology, Central South University, Changsha, China; ^2^ Aier Eye Institute, Changsha, China; ^3^ Key Laboratory for Regenerative Medicine of Ministry of Education, Jinan University, Guangzhou, China; ^4^ Key Laboratory of Optoelectronic Information and Sensing Technologies of Guangdong Higher Educational Institutes, Jinan University, Guangzhou, China

**Keywords:** keratocytes, polydimethylsiloxane (PDMS), spheroid culture, reprogramming, mechanotransduction

## Abstract

Suspended spheroid culture using ultralow attachment plates (ULAPs) is reported to effect corneal fibroblast reprogramming. Polydimethylsiloxane (PDMS), with hydrophobic and soft substrate properties, facilitates adherent spheroid formation that promotes cellular physical reprogramming into stem-like cells without using transcription factors. However, it is still unknown whether the biophysical properties of PDMS have the same effect on adult human corneal keratocyte reprogramming. Here, PDMS and essential 8 (E8) medium were utilized to culture keratocyte spheroids and fibroblast spheroids, and the reprogramming results were compared. We provide insights into the probable mechanisms of the PDMS effect on spheroids. qPCR analysis showed that the expression of some stem cell marker genes (*OCT4, NANOG, SOX2, KLF4, CMYC*, *ABCG2* and *PAX6*) was significantly greater in keratocyte spheroids than in fibroblast spheroids. The endogenous level of stemness transcription factors (*OCT4, NANOG, SOX2, KLF4* and *CMYC*) was higher in keratocytes than in fibroblasts. Immunofluorescence staining revealed Klf4, Nanog, Sox2, ABCG2 and Pax6 were positively stained in adherent 3D spheroids but weakly or negatively stained in adherent 2D cells. Furthermore, *OCT4*, *NANOG*, *SOX2*, *KLF4*, *HNK1*, *ABCG2* and *PAX6* gene expression was significantly higher in adherent 3D spheroids than in adherent 2D cells. Meanwhile, *SOX2*, *ABCG2* and *PAX6* were more upregulated in adherent 3D spheroids than in suspended 3D spheroids. The RNA-seq analysis suggested that regulation of the actin cytoskeleton, TGFβ/BMP and HIF-1 signaling pathways induced changes in mechanotransduction, the mesenchymal-to-epithelial transition and hypoxia, which might be responsible for the effect of PDMS on facilitating reprogramming. In conclusion, compared to corneal fibroblasts, keratocytes were more susceptible to reprogramming due to higher levels of endogenous stemness transcription factors. Spheroid culture of keratocytes using PDMS had a positive impact on promoting the expression of some stem cell markers. PDMS, as a substrate to form spheroids, was better able to promote reprogramming than ULAPs. These results indicated that the physiological cells and culture conditions herein enhance reprogramming. Therefore, adherent spheroid culture of keratocytes using PDMS is a promising strategy to more safely promote reprogramming, suggesting its potential application for developing clinical implants in tissue engineering and regenerative medicine.

## Introduction

Keratocytes, the physiological resident cells in the corneal stromal layer, are derived from the neural crest (Johnston et al., 1979) and responsible for maintaining the integrity of the fibrils and the extracellular matrix (ECM) (Brunette et al., 2017). Keratocytes are quiescent and express ALDH3A1, lumican and keratocan in the physical environment. When keratocytes are removed from stromal tissue and cultured in serum-containing medium, they exhibit the morphological characteristics of fibroblasts or myofibroblasts and express fibronectin and α-SMA (Kumar et al., 2016). It is well known that corneal fibroblasts can be successfully reprogrammed to form induced pluripotent stem cells (iPSCs) by transfecting with transcription factor genes (OSKM: *Oct4, Sox2, Klf4, c-Myc*) (Bikkuzin et al., 2019). However, the reprogramming of corneal keratocytes has not yet been investigated. Of note, some physiological cells, such as hepatocytes, gastric epithelial cells and keratinocytes, appeared to be more easily reprogrammed and required fewer retroviral integrations than fibroblasts (Aasen et al., 2008; Aoi et al., 2008). Therefore, it is worthwhile to investigate whether a difference between physiological keratocytes and fibroblasts exists in the cornea.

Spheroid culture, as a traditional strategy, is generally used for various cell types, including human corneal endothelial cells (CECs) (Mimura et al., 2010), human adipose-derived stem cells (hADSCs) (Cheng et al., 2013; Guo et al., 2015), human iPSCs (hiPSCs) (Haraguchi et al., 2015) and retinal cells (Coles et al., 2004). Spheroid culture enhances the keratocyte phenotype of corneal stromal cells (Funderburgh et al., 2008; Li et al., 2015) and promotes the expression of stem cell markers (e.g., CD34, Nestin and Nanog) (Lai and Tu, 2012) or other specific markers (e.g., BMP3 and cadherin 5) (Scott et al., 2011). In addition, a previous study reported that spheroid culture also affected corneal stromal cell reprogramming, which promoted the expression of stemness transcription factors (e.g., Nanog and Oct4) (Byun et al., 2014). Moreover, Higgins et al. (2013) suggested that partial reprogramming was enabled by the three-dimensional (3D) spheroid culture of papilla cells, which were undergoing cell intrinsic reprogramming, in part by generating their own ECM or growth factors and enhancing/enabling intercellular communication.

There are several common methods to induce cellular spheroid formation, such as using ultralow attachment plates (Byun et al., 2014), agarose 3D Petri dishes (Guo et al., 2015), chitosan films (Cheng et al., 2012) and methylcellulose (Lai and Tu, 2012). Polydimethylsiloxane (PDMS), the hydrophobic ECM surface, provides a soft substrate microenvironment that facilitates spheroid formation and increases the reprogramming efficiency of mesenchymal stem/stromal cells (MSCs) into iPSCs (Gerardo et al., 2019). Guo and colleagues (Guo et al., 2014) reported that HEK-293 cells were reprogrammed into stem-like cells by spherical culture, in combination with serum-containing media, on a low-adhesion soft PDMS substrate without using transcription factors. They found that the key marker proteins of stem cells, transcription factors OCT4 and Nanog, were significantly upregulated in HEKs cultured on soft PDMS, whereas neurofilament-M, a native HEK marker, was downregulated, emphasizing the critical role of physical cues in reprogramming. Because reprogramming transcription factors requires integrated viral DNA and can be complicated by oncogenes, the Guo study provided a safe way to achieve cellular reprogramming without the viral and tumorigenesis risks. However, it is still unknown whether the biophysical properties of PDMS have the same effect on adult human corneal keratocyte reprogramming. Hence, the effect of PDMS on facilitating reprogramming of keratocyte spheroids is worthy of study, which is beneficial to its potential application in producing clinical implants in tissue engineering and regenerative medicine.

In the current study, we acquired and subcultured human corneal keratocytes and fibroblasts from small incision lenticule extraction (SMILE)-derived lenticules using different types of culture media. Then, we utilized PDMS and essential 8 (E8) medium to culture adherent 3D keratocyte spheroids and fibroblast spheroids and compared their reprogramming results. Finally, we provide insights into probable mechanisms of the PDMS effect on adherent spheroids using RNA-Seq analysis. Our study provides a biophysical approach without the application of any exogenous genes, RNAs or proteins, which may aid in the discovery of a safer strategy to achieve cellular reprogramming.

## Materials and Methods

### Ethics Statement

The use of human tissue samples was approved by the ethics committee of Aier Eye Hospital (Changsha, Hunan, China) (AIER2018IRB24), and the methods for securing human tissues were in compliance with the Declaration of Helsinki. Informed consent was obtained from all patients.

### Isolation and Culture of Human Corneal Stromal Cells

Human corneal stromal cells (hCSCs) were isolated from SMILE-derived lenticules (Changsha Aier Eye Hospital, Changsha, China). The lenticules were rinsed in sterile PBS and digested with 1 mg/ml of collagenase I (Sigma-Aldrich, St. Louis, MO, United States) for 6–8 h at 37°C. The mixture was then centrifuged to collect the cells. After centrifugation, the pellets were separately cultured in soluble porcine corneal stromal extract (pCSE) with low-serum RIFA medium (RIFA + pCSE) (Li et al., 2021), containing DMEM/F12 (Gibco, Gaithersburg, MD, United States), 5 μg of protein/ml of soluble pCSE, 10 μM of ROCK inhibitor Y27632 (AdooQ Bioscience, Irvine, CA, United States), 10 ng/ml of ITS (Life Technologies, Carlsbad, CA, United States), 10 ng/ml of FGF2 (PeproTech, Rocky Hill, NJ, United States), 1 mML-ascorbate 2-phosphate (Sigma, St. Louis, MO, United States), 0.5% FBS (Gibco, Gaithersburg, MD, United States) and 1% penicillin-streptomycin (P/S, Gibco, Gaithersburg, MD, United States), or in normal medium (NM) containing DMEM/F12, 10% FBS and 1% penicillin-streptomycin. The cells were seeded on collagen-coated cell plates at a concentration of 4 × 10^4^ cells/ml. The plates were incubated at 37°C in a humidified atmosphere at 5% CO_2,_ and the media were changed every 2–3 days. Confluent cell layers were dissociated and subcultured at the same seeding density with RIFA + pCSE or NM. Only hCSCs at passage two (P2) were used in this study.

### Preparation of Polydimethylsiloxane Culture Substrates

Sylgard 184 (Dow Corning, United States) was prepared by mixing 10 parts of the base with 1 part of the curing agent for 20 min at 500 RPM in a magnetic stirrer followed by a defoaming cycle of 5 min at 2000 RPM. PDMS formulations were coated onto 6-well tissue culture plates (TCPs, Corning Costar, United States) to create 1.5 mm thick films followed by curing at 70°C for 8 h. The PDMS plates were sterilized with UV light for 1 h in a laminar flow hood, and some plates were coated using FNC Coating Mix (AthenaES^®^, United States) to promote cell adhesion for further use.

### Contact Angle Measurement and Spherical Formation Assay

The contact angles of TCPs, PDMS and FNC-coated PDMS were measured by a goniometer (Drop Shape Analysis System, KRUSS, Germany). For each of the measurements, a 50 µL water drop was generated on the surface using the commercial instrument at room temperature. Drop image was taken simultaneously by the camera of commercial instrument with perpendicular perspectives to each other. Contact angle measurements were performed three times at different locations on each surface.

After sterilization with UV light for 1 h, keratocytes were seeded and cultured on TCPs, PDMS and FNC-coated PDMS at the same cell density using RIFA + pCSE. The cells were incubated at 37°C in a humidified atmosphere at 5% CO_2_ for 2 days.

### Spheroid Culture of Keratocytes and Fibroblasts Using PDMS and E8 Medium

The culture conditions were prepared with reference to published papers (Byun et al., 2014; Guo et al., 2014; Ghoubay-Benallaoua et al., 2017; Gerardo et al., 2019). Briefly, after establishing and expanding adherent cultures, the keratocytes at P2 were digested by Accutase (Millipore, United States) and were separately transferred to 6-well ultralow attachment plates, 6-well PDMS plates, and 6-well TCPs (2 × 10^6^ cells/well) in RIFA + pCSE for 1–2 days followed by essential 8 (E8) medium (Gibco, United States) for 6–7 days. Fibroblasts at P2 were also cultured in 6-well PDMS plates in the same way. The cells were incubated at 37°C in a humidified atmosphere at 5% CO_2_ for spheroid culture. The medium was changed every other day for 7–9 days.

### Cell Culture With Small Molecules

In cell culture experiments with small molecules, cells were cultured with or without 10 μM blebbistatin (Millipore, United States) for 7 days. The medium was changed every other day. The cells were protected from light during small molecule treatment.

### Quantitative Polymerase Chain Reaction

Total RNA from cells was extracted using the High Pure RNA Isolation Kit (Roche, Switzerland). cDNA was synthesized using a RevertAid First Strand cDNA Synthesis Kit (Thermo Scientific, United States) according to the manufacturer’s instructions. Gene-specific primers were synthesized by TsingKe Biotech (China), and the sequences of the primers are listed in Supplementary Table S1. For qPCR experiments, gene expression was analyzed by qPCR (Roche, Switzerland) with three replicates per sample. The amplification results were normalized to GAPDH mRNA transcript levels. Expression changes in the gene transcripts for each sample were calculated using the 2^−△△Ct^ method. The results from three independent experiments were statistically analyzed.

### Immunofluorescence Staining

After the cells were fixed, permeabilized and blocked, they were incubated overnight at 4°C with primary antibodies, including rabbit anti-Lumican (1:500, Abcam, United Kingdom), rabbit anti-Fibronectin (1:200, Abcam, United Kingdom), rabbit anti-α-SMA (1:500, Abcam, United Kingdom), rabbit anti-Nanog (1:100, Abcam, United Kingdom), rabbit anti-Sox2 (1:250, Abcam, United Kingdom), mouse anti-Oct4 (1:100, CST, United States), rabbit anti-Klf4 (1:100, Abcam, United Kingdom), mouse anti-ABCG2 (1:50, Abcam, United Kingdom), rabbit anti-Pax6 (1:50, Abcam, United Kingdom), rabbit anti-Myosin Ⅱa (1:50, CST, United States), rabbit anti-HIF1α (1:800, CST, United States) and rabbit anti-YAP1 (1:200, Bioss, China) followed by washing three times in PBS and incubation with FITC-conjugated anti-rabbit or anti-mouse IgG secondary antibodies (1:500, Life, United States) for 1 h at room temperature. Cells were then incubated with DAPI for nuclear staining for 15 min. Finally, imaging was performed using an LSM800 confocal microscope (Zeiss, Germany). The fluorescence intensity was quantified using ImageJ software (version 1.47, National Institutes of Health, United States).

### Western Blot

Total proteins were extracted, and the protein concentrations were detected using a BCA Protein Assay Kit (Solarbao, China). The protein samples were resolved by sodium dodecyl sulfate-polyacrylamide gel electrophoresis (SDS-PAGE) and transferred to polyvinylidene fluoride (PVDF) membranes. After blocking, the membranes were incubated with antibodies, including rabbit anti-ALDH3A1 (1:500, Abcam, United Kingdom), rabbit anti-keratocan (1:100, Abcam, United Kingdom), rabbit anti-lumican (1:1,000, Abcam,VUK), rabbit anti-fibronectin (1:1,000, Abcam, United Kingdom) and mouse anti-GAPDH overnight at 4°C. The membranes were then incubated with anti-mouse or anti-rabbit IRDye 680RD secondary antibodies (1:10,000, LI-COR Biosciences, United States) for 1 h at room temperature. The protein bands were visualized with the Odyssey Fc Imaging System (LI-COR Biosciences, United States) and quantified with ImageJ software. The expression ratios of the target proteins were determined after normalizing to the GAPDH levels. The results from three independent experiments were statistically analyzed.

### RNA-Seq Analysis

Total RNA was extracted from the suspension 3D, adherent 3D and adherent 2D groups on day 7 using TRIzol reagent. RNA-Seq was subcontracted to Chi Biotech Co., Ltd. (Shenzhen, China). To obtain normalized gene expression levels, we calculated the number of reads per kilobase of exon model per million mapped reads (RPKM) after library construction and sequencing. The original RNA-seq was mapped to the reference transcriptome sequence using FANSe2 as previously described (Xiao et al., 2014). The correlation coefficients between gene expression levels were calculated and plotted as a correlation heat map. The screening threshold for the differentially expressed genes (DEGs) was set to |log2 (fold change)| > 1 and *p* < 0.05. topGO software (version 2.18.0) was used to perform gene ontology (GO) analysis, and comparisons between two groups were made using Fisher’s exact test. Pathway enrichment analysis was primarily based on the Kyoto Encyclopedia of Genes and Genomes (KEGG) database using KOBAS software (kobas2.0-20150126), and comparisons between two groups were made using the hypergeometric test.

### Statistical Analysis

Values are expressed as the mean ± SD of values obtained from three or more samples. Statistical analysis between two groups was carried out using Student’s unpaired t-test; comparisons among multiple groups were determined by one-way ANOVA. *p* < 0.05 was considered to be statistically significant.

## Results

### Cultivation and Characteristics of Human Corneal Stromal Cells From SMILE-Derived Lenticules

**FIGURE 1:**
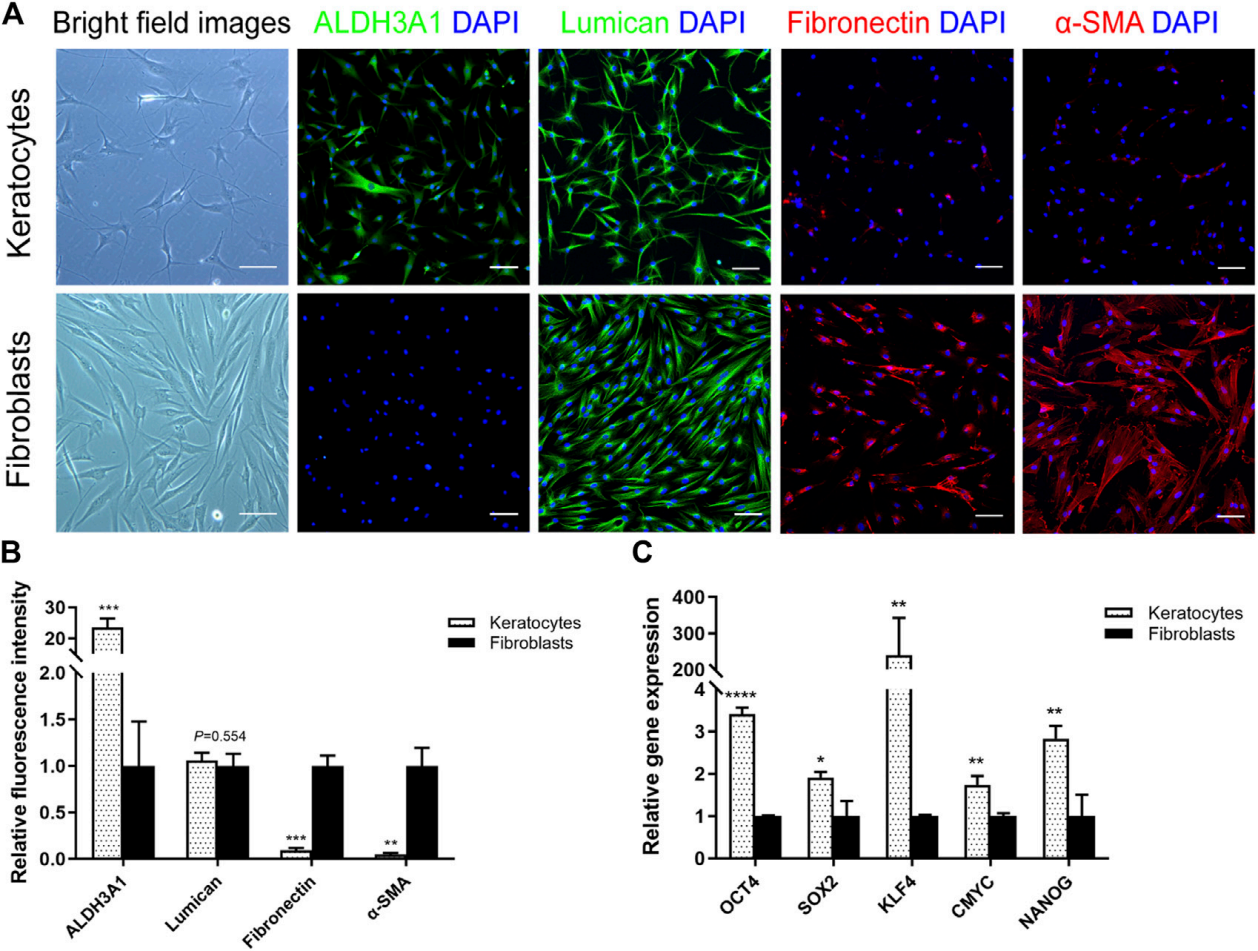
Differences between keratocytes and fibroblasts. **(A)** Light microscopy revealed that the hCSCs at P2 cultured in soluble porcine corneal stromal extract (pCSE) with low-serum RIFA medium (RIFA + pCSE) or normal medium (NM) had different morphologies. Immunofluorescence images of ALDH3A1, lumican, fibronectin, and α-SMA are shown. **(B)** Marker expression was represented as average ± S.D. from three independent experiments. **(C)** The qPCR results showed that the endogenous levels of *OCT4*, *SOX2*, *KLF4*, *CMYC* and *NANOG* in keratocytes were higher than those in fibroblasts. (*p < 0.05; **p < 0.01; ***p < 0.001; ****p < 0.0001). Scale bars: 100 μm.

To acquire primary hCSCs, hCSCs were isolated from SMILE-derived lenticules using collagenase I digestion and cultured in two kinds of culture medium, RIFA + pCSE and NM. The primary hCSC monolayer reached confluence within 5–7 days in RIFA + pCSE. The cells exhibited dendritic or stellate shapes, and cell processes extended to connect with neighboring cells. After subculturing in RIFA + pCSE, immunofluorescence analysis revealed that hCSCs at passage 2 (P2) strongly expressed keratocyte markers (ALDH3A1, lumican) and were weakly stained with (myo)fibroblast markers (fibronectin and α-SMA). In contrast, primary hCSCs exhibited spindle shapes in NM. The hCSCs at P2 in NM strongly expressed fibronectin and α-SMA (Figure 1A). Signal quantification also supported the distinctive expression pattern of markers in keratocytes and fibroblasts (Figure 1B). In addition, the endogenous levels of *OCT4, SOX2, KLF4, CMYC* and *NANOG* in keratocytes were higher than those in fibroblasts (3.41-, 1.91-, 239.6-, 1.74- and 2.83-fold, respectively; *p* < 0.05) (Figure 1C). The results indicate that the cells isolated from SMILE-derived lenticules have keratocyte morphological and phenotypic characteristics in RIFA + pCSE or become fibroblasts undergoing fibrosis in NM.

### Culturing Human Corneal Stromal Cells on Polydimethylsiloxane

**FIGURE 2:**
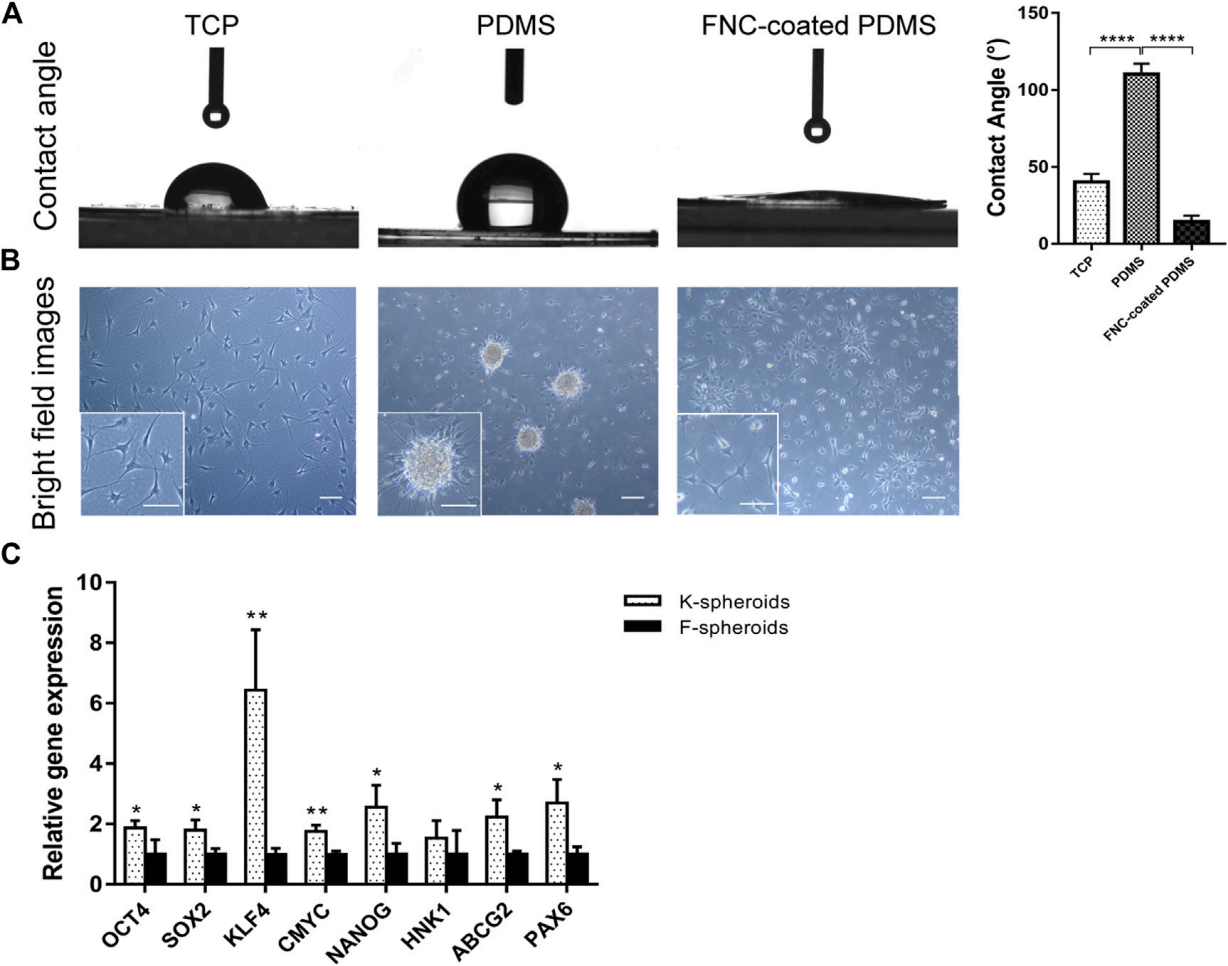
Characteristics of different spheroids and contact angles on TCPs, PDMS and FNC-coated PDMS. **(A)** The contact angle of PDMS was significantly higher than that of TCPs and FNC-coated PDMS. **(B)** Light microscopy revealed that keratocytes spontaneously formed spheroids on PDMS. The cells adherent to TCPs and FNC-coated PDMS without forming spheroids exhibited dendritic or stellate shapes, and cell processes extended to connect with neighboring cells. **(C)** qPCR analysis indicated that stemness transcription factors (*OCT4*, *NANOG*, *SOX2*, *KLF4* and *CMYC*) and corneal stem cell markers (*ABCG2* and *PAX6*) were significantly higher in keratocyte spheroids (K-spheroids) than in fibroblast spheroids (F-spheroids). (*p < 0.05; **p < 0.01; ***p < 0.001; ****p < 0.0001). Scale bars: 100 μm.

Hydrophobic PDMS was utilized to generate adherent 3D spheroid cultures of hCSCs from SMILE-derived lenticules. To create low attachment surface substrates, we coated tissue culture plates with 1.5 mm thick PDMS film. The contact angle of PDMS (110.3° ± 6.8°) was significantly higher than those of TCPs (40.6° ± 5.1°) and FNC-coated PDMS (14.3° ± 4.1°) (Figure 2A), which exhibited the hydrophobicity of PDMS. Light microscopy revealed that keratocytes spontaneously aggregated to form 3D spheroids on PDMS in RIFA + pCSE. The keratocyte spheroids gradually enlarged and stabilized at a diameter of 100 μm after 2 days of culture. Similar to the cellular morphology of the adherent keratocytes on TCPs, cells adherent to FNC-coated PDMS were unable to form spheroids in RIFA + pCSE. The adherent keratocytes exhibited dendritic or stellate shapes, and cell processes extended to connect with neighboring cells in the RIFA + pCSE group (Figure 2B). To obtain a better effect of forming spheroids, keratocytes and fibroblasts at P2 were cultured on PDMS using essential 8 (E8) medium. We found that keratocytes or fibroblasts formed adherent spheroids on PDMS in E8 medium. After culture on PDMS at day 7, keratocyte-adherent spheroids (K-spheroids) exhibited significantly higher expression of stem cell marker genes (*OCT4, NANOG, SOX2, KLF4, CMYC*, *ABCG2* and *PAX6*) than fibroblast-adherent spheroids (F-spheroids) (1.87-, 2.56-, 1.80-, 6.44-, 1.75-, 2.23- and 2.70-fold, respectively; *p* < 0.05) by qPCR analysis (Figure 2C). Based on these results, hCSCs enable the spontaneous and stable formation of spheroids adherent to the hydrophobic surface of PDMS in E8 medium. Additionally, keratocytes are more susceptible to reprogramming to obtain stemness in spheroid culture than corneal fibroblasts.

### Characteristics of Adherent Keratocyte 3D Spheroids and Adherent 2D Keratocytes

**FIGURE 3:**
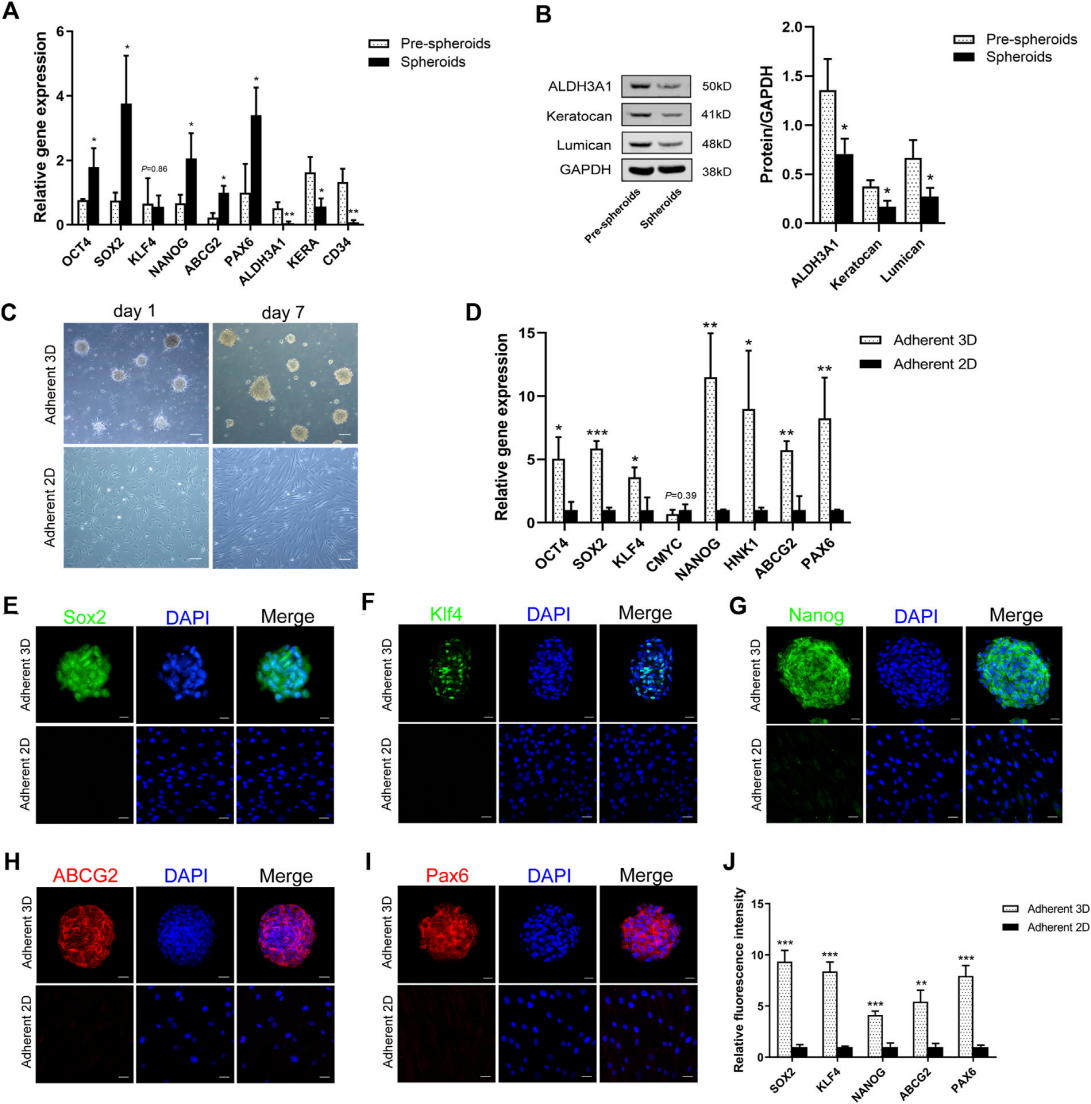
Characteristics of adherent 3D spheroids. **(A)** The qPCR results showed that stemness transcription factors (*OCT4*, *NANOG* and *SOX2*) and corneal stromal stem cell markers (*ABCG2* and *PAX6*) were upregulated, while the keratocyte markers (*ALDH3A1*, *KERA* and *CD34*) were downregulated in spheroids compared to keratocytes (pre-spheroids). **(B)** Western blotting confirmed that ALDH3A1, keratocan, and lumican were significantly less expressed in spheroids. **(C)** Light microscopy revealed the morphologic differences between adherent 3D spheroid cells and adherent 2D cells. **(D)** The qPCR results showed that *OCT4*, *SOX2*, *KLF4*, *NANOG*, *HNK1*, *ABCG2* and *PAX6* were upregulated in the adherence 3D group compared with the adherence 2D group. There was no significant difference in the expression of *CMYC* between the two groups. **(E–I)** Immunofluorescence images for Sox2, Klf4, Nanog, ABCG2 and Pax6 are shown. **(J)** Marker expression is presented as the average ± S.D. from three independent experiments. (*p < 0.05; **p < 0.01; ***p < 0.001). Scale bars: 100 μm for C; 20 μm for **(E–I)**.

To investigate the characteristics of keratocytes cultured on PDMS, we compared adherent 3D spheroids on PDMS (adherent 3D) with keratocytes (pre-spheroids) or adherent 2D cells on TCPs (adherent 2D) in E8 medium using immunofluorescence staining, qPCR and WB. Compared with keratocytes, keratocyte marker genes (*ALDH3A1*, *KERA* and *CD34*) were downregulated (0.12-, 0.34- and 0.06-fold, respectively; *p* < 0.05), while stemness markers (*OCT4*, *NANOG*, *SOX2*, *ABCG2* and *PAX6*) were upregulated (2.33-, 3.09-, 4.97-, 4.47- and 3.40-fold, respectively; *p* < 0.05) in spheroids (Figure 3A). Western blotting confirmed that ALDH3A1, keratocan, and lumican were significantly downregulated in spheroids (0.52-, 0.44-, and 0.41-fold, respectively; *p* < 0.05) (Figure 3B). Furthermore, compared with the adherent 2D group, most cells first spontaneously formed soft spheroids adhering to the PDMS surface in the adherent 3D group. Some individual cells attached to PDMS and surrounded spheroids. After 2 days of culture, the cells surrounding spheroids gradually disappeared, while spheroids became compact and remained adherent to the surface of PDMS. In the adherent 2D group, when cultured in E8 medium, the cellular morphology of adherent keratocytes gradually changed from dendritic or stellate shapes to long spindle shapes (Figure 3C). qPCR analysis revealed that stemness genes (*OCT4, NANOG*, *SOX2*, *KLF4*, *HNK1*, *ABCG2* and *PAX6*) were upregulated in the adherent 3D group compared with the adherent 2D group (5.06-, 11.48-, 5.85-, 3.59-, 8.99-, 5.73- and 7.58-fold, respectively; *p* < 0.05). There was no significant difference in the expression of *CMYC* between the two groups (*p* = 0.39) (Figure 3D). In addition, immunofluorescence staining also showed that adherent 3D spheroids at day 7 were stained positive for Sox2, Klf4, Nanog, ABCG2 and Pax6, whereas adherent 2D cells exhibited negative or weak staining. Signal quantification also supported the distinctive expression pattern of markers in both spheroids and adherent cells (Figure 3J). Taken together, these results indicate that the spheroid culture of keratocytes on the surface of PDMS has a positive impact on promoting the expression of stemness markers (Klf4, Nanog, Sox2, ABCG2 and Pax6) and reducing native keratocyte markers (ALDH3A1, keratocan, and lumican).

### Transcriptome Profiles of Keratocyte Spheroids on Polydimethylsiloxane

**FIGURE 4:**
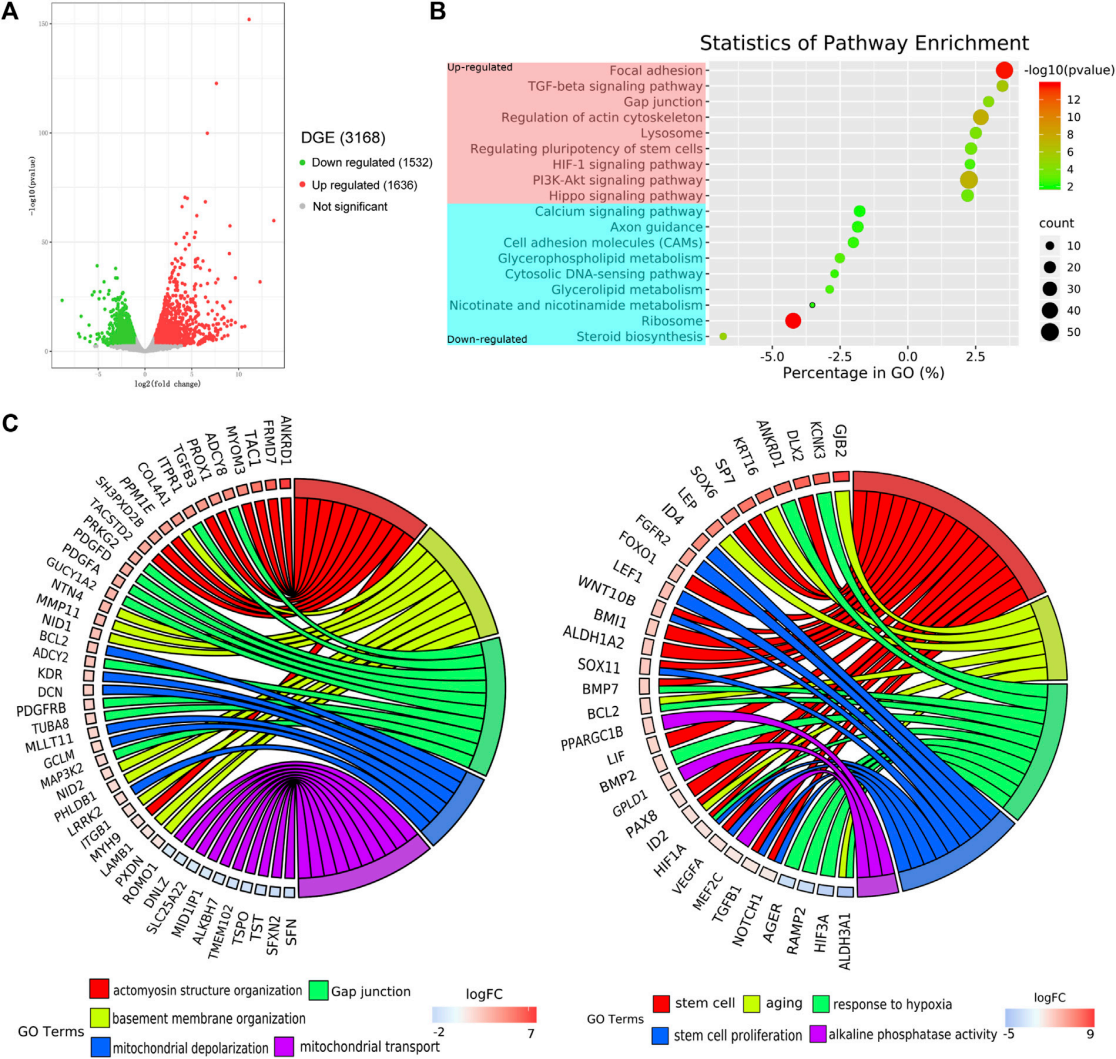
Transcriptome profiles of adherent 3D keratocyte spheroids on PDMS. **(A)** The volcano plots show the number of differentially expressed genes (DEGs) in the adherence 3D group and adherence 2D group. **(B)** Through Kyoto Encyclopedia of Genes and Genomes (KEGG) analysis, the most significantly changed signaling pathways in the adherence 3D group compared with the adherence 2D group are shown. **(C)** The relationship between genes and significant gene ontology (GO) terms are listed by GO chords.

**FIGURE 5:**
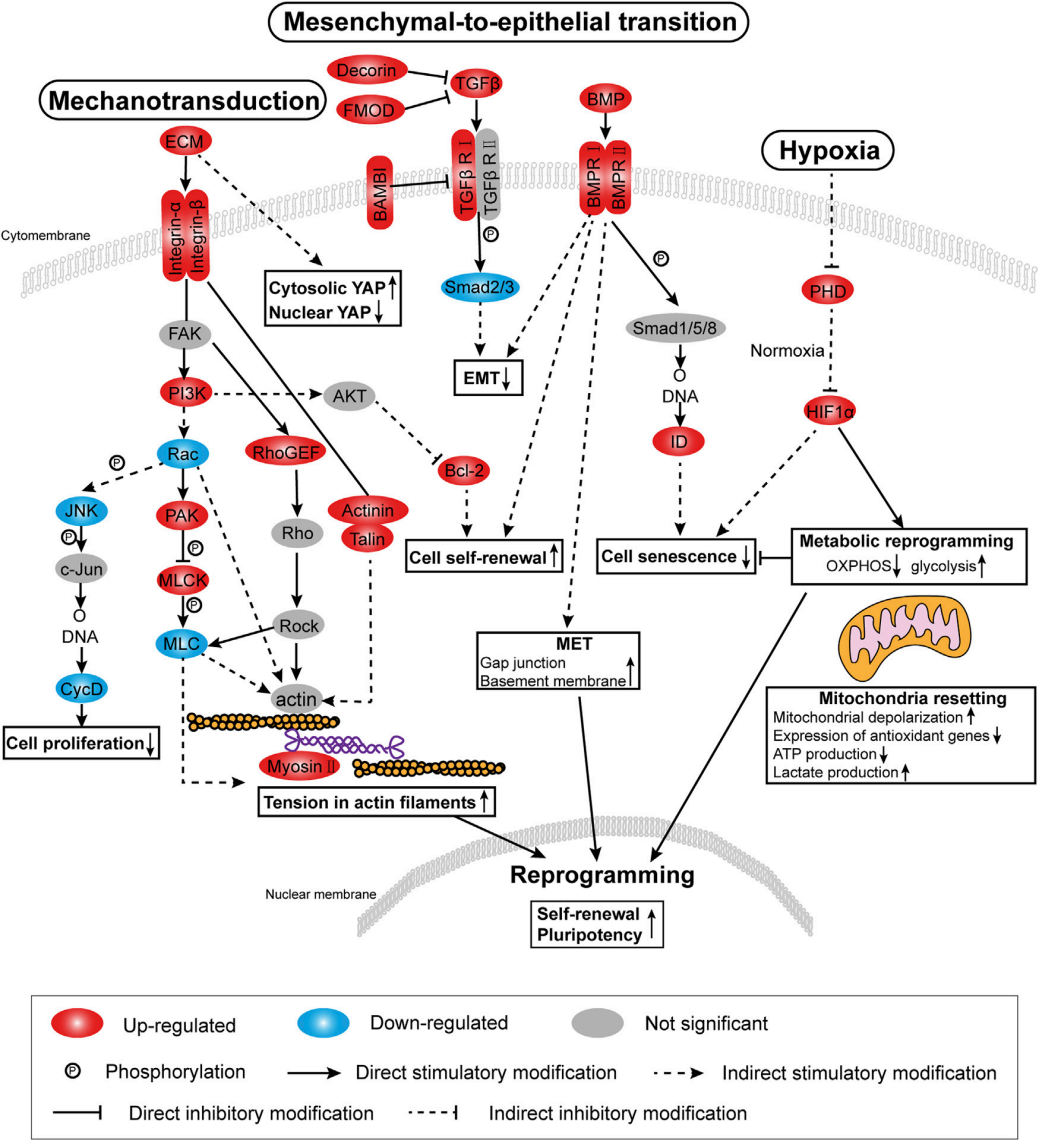
Signaling pathway diagram based on RNA-seq analysis. According to DEGs, the KEGG mapper database and the literature, the diagram associated with facilitating reprogramming highlights mechanotransduction-related, mesenchymal-to-epithelial transition (MET)-related and hypoxia-related signaling pathways.

In this analysis, the screening threshold for the differentially expressed genes (DEGs) was set to |log2 (fold change)| > 1 and *p*-Value < 0.01. RNA-seq analysis identified 3,168 DEGs in the adherent 3D group compared with the adherent 2D group, with 1,636 upregulated genes and 1,532 downregulated genes. The volcano plots showed the number of DEGs in these two groups (Figure 4A). Through Kyoto Encyclopedia of Genes and Genomes (KEGG) analysis, 18 of the most significantly changed signaling pathways in the adherent 3D group compared with the adherent 2D group were shown, including focal adhesion, HIF-1, calcium, and ribosome signaling pathways (Figure 4B). The GO term enrichment histograms showed that in the adherent 3D group compared with the adherent 2D group, the stem cell-, actin cytoskeleton-, ECM-, focal adhesion-, aging-, hypoxia-, cell proliferation-, mitochondrion-, gap junction- and alkaline phosphatase-related GO terms were upregulated, whereas the mitochondrial transport-, tricarboxylic acid transport- and senescence-related GO terms were downregulated (Supplementary Figure S1). Some key GO terms, such as stem cells, aging, response to hypoxia, stem cell proliferation, alkaline phosphatase activity, actomyosin structure organization, basement membrane organization, gap junction, mitochondrial depolarization and mitochondrial transport, were listed by GO chords (Figure 4C). Bioinformatic analysis was performed using the KEGG database to predict the probable mechanism of PDMS facilitating reprogramming. According to the DEGs, KEGG Mapper database and literature, the signaling pathway diagram associated with reprogramming was plotted (Figure 5). This figure shows the position of key DEGs in the reprogramming-associated signaling pathway network in the adherent 3D group compared to the adherent 2D group. The inferred schematic displays the mechanotransduction-, mesenchymal-to-epithelial transition (MET)- and hypoxia-related signaling pathways induced by PDMS, which might be responsible for the effect of PDMS on facilitating reprogramming.

**FIGURE 6:**
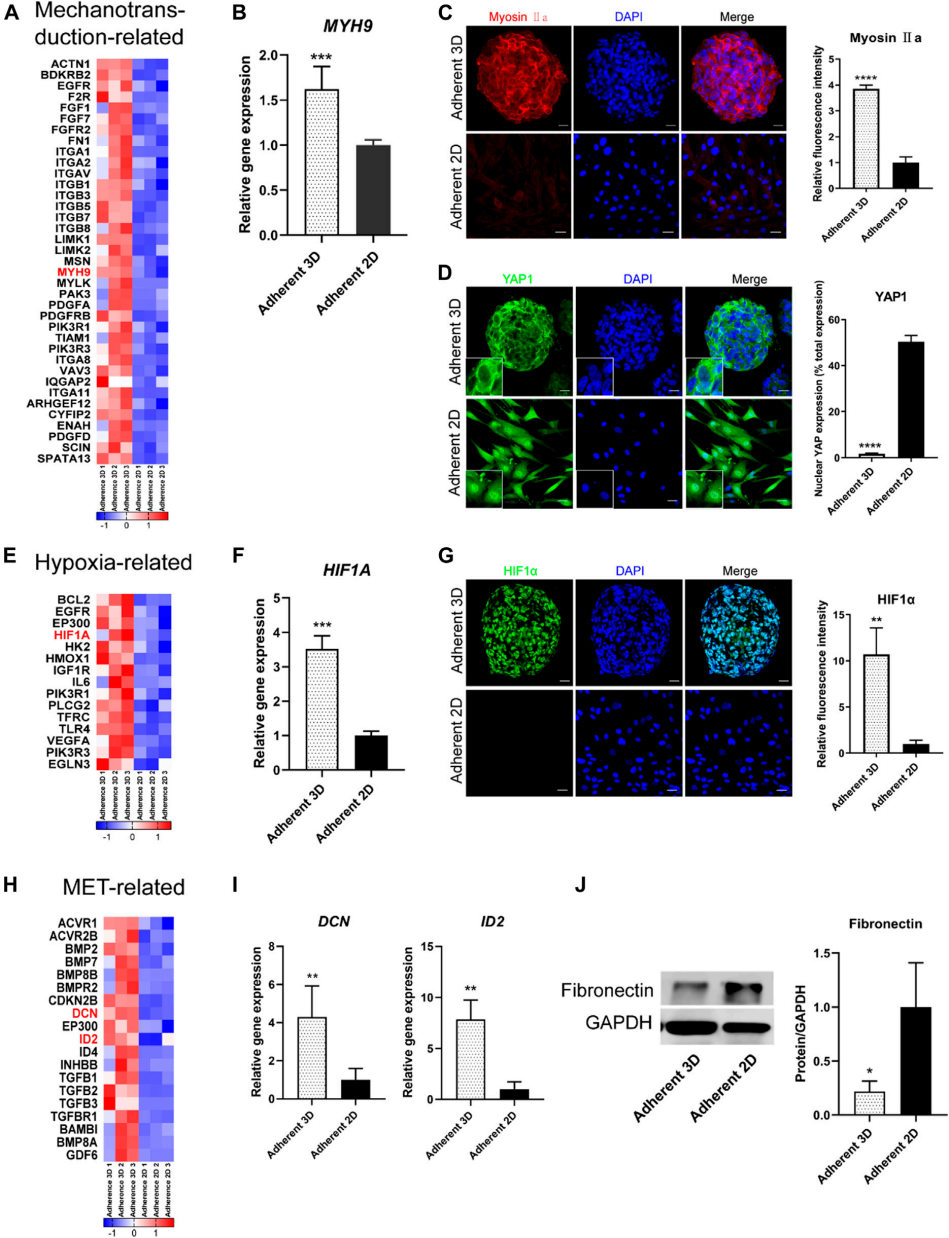
Analysis of the effects of PDMS on keratocyte spheroids. **(A,E,H)** The heat map shows the DEGs of mechanotransduction-related, hypoxia-related and MET-related signaling pathways. **(B–D,F–J)** PDMS acts on these three signaling pathways, which were validated by IF, qPCR and WB analyses. (*p < 0.05; **p < 0.01; ***p < 0.001; ****p < 0.0001). Scale bars: 20 μm for **(C,D,G)**.

Next, genes were selected from the above RNA-seq data and the signaling pathways to obtain heat maps (Figures 6A,E,H). To validate the signaling pathway diagram, we confirmed several DEGs by qPCR, Western blot and immunofluorescence staining. qPCR analyses showed that mechanotransduction-related genes (*MYH9*), hypoxia-related genes (*HIF1A*) and MET-related genes (*DCN* and *ID2*) were significantly more abundant in the adherent 3D group than in the adherent 2D group (Figures 6B,F,I). Immunofluorescence staining showed that the mechanotransduction-related protein (Myosin Ⅱa) and hypoxia-related protein (HIF1α) were strongly stained in the adherent 3D group but weakly or negatively stained in the adherent 2D group (Figures 6C,G). In addition, cytoplasmic staining of YAP was mainly observed with weak nuclear staining in the adherent 3D group, whereas localization of YAP was strongly positive in the nuclei of the adherent 2D group (Figure 6D). These differences in expression were significant (*p* < 0.01). Western blot analyses also showed that the EMT-related marker fibronectin was significantly expressed at lower levels in the adherent 3D group (Figure 6J). Meanwhile, to explore the different characteristics between adherent 3D spheroids and suspended 3D spheroids, keratocytes cultured in E8 medium were divided into three groups: the suspension 3D group, adherent 3D group and adherent 2D group. qPCR analyses showed that *SOX2, ABCG2* and *PAX6* were significantly higher in the adherent 3D group than in the suspension 3D group (4.95-, 7.91- and 3.54-fold; *p* < 0.01), whereas *OCT4, NANOG* and *KLF4* were not significantly different in these two groups (Supplementary Figure S2).

**FIGURE 7:**
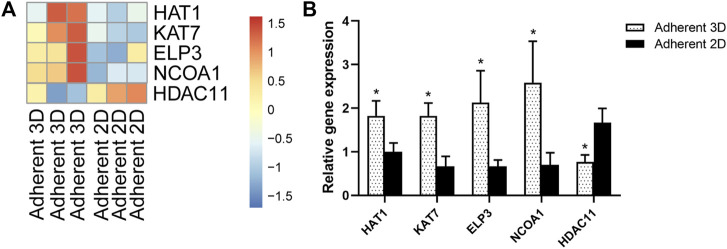
Mechanotransduction increased expression of histone acetyltransferase genes and reduced expression of histone deacetylase genes. **(A)** The heat map shows the DEGs of histone acetyltransferase and histone deacetylase. **(B)** Mechanotransduction acts on histone, which was validated by qPCR. (*p < 0.05).

To explore the cells’ epigenetic state in this study, a series of histone acetyltransferase genes (*HAT1*, *KAT7*, *ELP3* and *NCOA1*) and histone deacetylase genes (*HDAC11*) were selected from the above RNA-seq data and were validated by qPCR. The heat maps showed that, compared with adherent 2D group, *HAT1*, *KAT7*, *ELP3* and *NCOA1* were up-regulated, whereas *HDAC11* was down-regulated in adherent 3D group (Figure 7A). qPCR results also showed that *HAT1*, *KAT7*, *ELP3* and *NCOA1* were significantly higher in the adherent 3D group than in the adherent 2D group (1.82-, 2.74-, 3.19- and 3.70-fold; *p* < 0.05), whereas *HDAC11* was down-regulated in adherent 3D group (0.46-fold; *p* < 0.05) (Figure 7B).

To gain deeper insight into the facilitation reprogramming role of mechanotransduction-related signaling pathways induced by PDMS, we inhibited Myosin Ⅱa by supplying the myosin II inhibitor Blebbistatin in suspended 3D spheroid cultures, adherent 3D spheroid cultures and adherent 2D cell cultures. The results showed that the expression levels of *SOX2, ABCG2* and *PAX6* in adherent 3D spheroids treated with blebbistatin were significantly lower than those in adherent 3D spheroids without blebbistatin treatment (*p* < 0.05). There was no significant difference in these genes after treatment with blebbistatin in the suspension 3D group, adherent 3D group or adherent 2D group (Supplementary Figure S3).

Based on the RNA-seq data and validation results, we speculate that the effect of PDMS on facilitating reprogramming may be related to regulation of the actin cytoskeleton, TGFβ/BMP, and/or the HIF-1 signaling pathway.

## Discussion

Since the discovery that somatic cells (murine fibroblasts) could be reprogrammed into induced pluripotent stem cells (iPSCs) in 2006, an increasing number of studies have explored a more appropriate reprogramming induction system and target cells to increase reprogramming efficiency. To date, a variety of cell types have been reported to be suitable for reprogramming, including blood cells (Chen et al., 2019), adult stem cells (Jiang et al., 2019) and even cancer cells (Bang et al., 2019). With respect to corneal stromal cells, most previous studies used reprogrammed corneal fibroblasts to generate iPSCs (Greene et al., 2014; Bikkuzin et al., 2019). Chien et al. (2012) reported that using the four-gene delivery method (Oct4/Sox2/Klf4/c-Myc), they successfully created iPSCs by dedifferentiating corneal keratocytes isolated from corneal tissues. Under a serum-free and feeder-free culture system, these keratocyte-reprogrammed iPSCs remained stable through 30 passages while retaining ESC-like pluripotency and accelerating corneal wound healing. Greene et al. (2016) found that keratocytes could be induced to produce collagen type II by phenotypic reprogramming with exogenous chondrogenic factors. These results showed that adult corneal keratocytes exhibited phenotypic plasticity, and all corneal stromal cells were susceptible to phenotypic reprogramming.

Cells cultured in spheroids grow with similar characteristics to cells *in vivo* and can simulate native tissue behaviors much more accurately than 2D cultures. Studies have already demonstrated that spheroid cultures promote cell stemness (Li et al., 2015). Moreover, cells in spheroids increase cell viability and functional performance compared with monolayer cultures (Lin and Chang, 2008). Conversely, the especially formulated E8 medium has been widely used for the growth and expansion of iPSCs (Reichman et al., 2017). This simplified, defined medium provides a much cleaner background for examining specific pathways in self-renewal, cell death, and differentiation and supports substantially improved reprogramming efficiencies (Chen et al., 2011). Investigators previously suggested that adult stromal stem cells (SSCs) cultured in E8 medium are characterized by sphere formation, expression of Pax6, Sox2, Bmi1, Nestin, ABCG2, Keratocan, Vimentin, Sox9, Sox10 and HNK1, production of collagen fibrils and differentiation into keratocytes, fibroblasts and myofibroblasts. It was previously shown that E8 medium allowed SSC-derived spheroids to grow with maintenance of stemness through 48 population doublings together with various differentiation potentials (Ghoubay-Benallaoua et al., 2017). Additionally, in combination with iPSC conditioned medium, increased expression of Nanog, Sox2 and Oct4 was also described to occur in mesenchymal stem/stromal cells cultured on PDMS when compared to a stiffer substrate, indicating that soft low-adhesion substrates favor full iPSC reprogramming (Gerardo et al., 2019).

Our results were consistent with previous studies, which showed that spheroid culture using PDMS and E8 medium facilitated reprogramming. Beyond that, our work revealed two major findings. First, we found that keratocytes were more susceptible to adherent 3D spheroid culture to obtain stemness than corneal fibroblasts. Second, several stem cell markers were more upregulated in adherent 3D spheroids than in adherent 2D cells and suspended 3D spheroids, but native keratocyte markers were downregulated.

The investigators found that hepatocytes, gastric epithelial cells and keratinocytes appeared to be more easily reprogrammed than fibroblasts (Aasen et al., 2008; Aoi et al., 2008). Aasen and colleagues (Aasen et al., 2008) speculated that endogenous transcription factor levels, the presence of stem cells in target cells and the epigenetic states of reprogrammed cells might be responsible for this difference. Consistent with these findings, we uncovered higher endogenous levels of *OCT4, SOX2, KLF4, CMYC* and *NANOG* in keratocytes than in fibroblasts, which may partly explain why spheroid cultures of keratocytes obtain stemness more easily than fibroblasts.

Spheroid formation in ULAPs, as a classical method to form suspended 3D spheroids, is widely utilized for many cell types (Ni et al., 2014; Bu et al., 2020). Byun et al. (2014) found that stemness transcription factor genes (*NANOG* and *OCT4*) were upregulated during suspended 3D spheroid culture in ULAPs. Nevertheless, the different characteristics of adherent 3D spheroids and suspended 3D spheroids are still unknown. Therefore, based on previous studies, we performed spheroid culture in both PDMS and ULAPs. Notably, several stemness genes in adherent 3D spheroids were significantly more highly expressed than those in suspended 3D spheroids.

Physiologically, keratocytes have a predominantly cortical distribution of stress fibers. These stress fibers, regulated by Rho signaling, are composed of actin and actin-associated proteins, including myosin II, which plays a critical role in creating contractile forces for controlling actomyosin contractility (Anderson et al., 2004). Meanwhile, myosin II forms actin filaments and creates force and tension because its motor domain binds to actin filaments (Asensio-Juárez et al., 2020). These actin filaments compose the cytoskeleton that bridges chromatin domains in the nucleus to the cell membrane and the extracellular matrix, providing a path for the well-known effects of mechanical stress on gene expression (Heisenberg and Bellaïche, 2013). In addition, Na et al. (2008) suggested that the cytoskeleton can transmit information (e.g., physical cues) much faster than chemical messengers between the cell exterior and interior, and similar to a tin can telephone, this communication requires tension in the linker. In our study, the myosin II gene *MYH9* was more highly expressed in adherent 3D spheroids, indicating the high tension of actin in PDMS-derived spheroids. After treatment with blebbistatin, the effect of PDMS on facilitating reprogramming was weakened. It was reported that the increased force in actin was essential to reprogramming and retaining stemness during spheroid culture upon softening PDMS (Guo et al., 2014). Thus, we presume that soft and hydrophobic PDMS can promote keratocyte reprogramming partially associated with the triggered mechanotransduction axis, which may explain why PDMS facilitates reprogramming better than ULAPs. Recently, the concept of mechanoepigenetics has been proposed to describe mechanisms involving force-induced physical changes to chromatin. Gerardo et al. (2019) demonstrated that mesenchymal stem/stromal cells (MSCs) cultured on soft substrates presented more relaxed nuclei, more euchromatic and less heterochromatic nuclear DNA regions, and increased expression of pluripotency-related genes, and these changes correlate with the reprogramming of MSCs. Downing et al. (2013) reported that microtopography elements (microgrooves) influence the epigenetic state of non-transduced cell chromatin and consequent reprogramming efficiency of mouse or human fibroblasts into iPSCs. Such mechanical cues led to increased histone H3 acetylation (AcH3) and methylation (H3K4me2 and H3K4me3) marks associated with transcriptional activation, through a mechanism that involves the decrease of histone deacetylase (HDAC) activity and upregulation of WD repeat domain 5 (WDR5) expression (a subunit of H3 methyltranferase). Similarly in our study, a series of histone acetyltransferase genes (*HAT1*, *KAT7*, *ELP3* and *NCOA1*) were up-regulated and histone deacetylase genes (*HDAC11*) was down-regulated in adherent 3D group. Therefore, it is possible that mechanotransduction induced by PDMS regulates reprogramming enhancement in adherent 3D keratocyte cultures partly through increased histone H3 acetylation.

MET can be considered a hallmark of the initiation phase of the reprogramming process and may be a fundamental cellular response that is required to reprogram a variety of cell types lacking epithelial characteristics and maintains their pluripotent state. Induction of MET was the major function of BMP signaling during the initiation phase of reprogramming (Samavarchi-Tehrani et al., 2010). According to the RNA-seq results, BMP signaling was activated. Meanwhile, epithelial-related genes were upregulated in PDMS-derived spheroids. Western blot assays also confirmed that TGFβ1-induced EMT was inhibited. These results demonstrate that spheroid culture on PDMS may activate the BMP-MET signaling axis, which has an important impact during the reprogramming initiation process.

During cellular reprogramming, somatic cells exhibit an increase in the rate of glycolysis and a reduction in oxidative phosphorylation (OXPHOS). Concomitantly, mitochondrial metabolism is decreased through the downregulation of mitochondrial genes and reduced mitochondrial density, leading to decreased oxygen consumption (Wu et al., 2016). Furthermore, hypoxia increases reprogramming efficiency by facilitating the metabolic transition necessary to sustain the energetic demands of the pluripotent state (Yoshida et al., 2009). In our study, hypoxia was generated during spherical culture. Compared with the TCP group, PDMS-derived spheroids exhibited a series of hypoxia-induced changes under normoxic conditions. Wu et al. (2016) suggested that the metabolic transition played a very active and dynamic role during cellular reprogramming to pluripotency and might even help drive the reprogramming process. In addition, hypoxia-inducible factor 1α (HIF1α) is known as a key regulator in responses to hypoxia. Kim et al. (2019) reported that HIF1α negatively regulated stem cell aging under hypoxia. However, they also found that HIF1α induced pluripotency- and glycolysis-related genes and repressed mitochondrial biogenesis, which reduced cellular damage and enhanced the potential for self-renewal and pluripotency. To the best of our knowledge, in addition to the mechanisms of mechanotransduction and MET, hypoxia may be another factor that plays an important role in promoting reprogramming by HIF1α.

**FIGURE 8:**
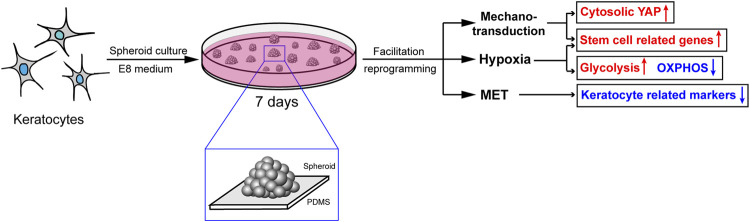
The probable mechanisms of the effect of PDMS on facilitating reprogramming. Mechanotransduction, MET and hypoxia induced by PDMS might be responsible for the promotion of keratocyte reprogramming.

Based on our RNA-seq results, we demonstrated that regulation of the actin cytoskeleton, TGFβ/BMP and HIF-1 signaling pathways induced changes in actin filament tension, MET and metabolism, respectively, which might explain the effect of PDMS on facilitating reprogramming (Figure 8).

Intriguingly, the corneal stromal stem cell markers ABCG2 and Pax6 were more highly expressed in the PDMS group than in the other two groups. Studies have shown that corneal epithelial stem cells grown on soft substrates are able to maintain limbal stem cell markers, whereas cells cultured on stiff substrates tend to differentiate by the Hippo-Yes-associated protein (YAP) signaling pathway (Foster et al., 2014; Gouveia et al., 2019). Muppala et al. (2019) suggested that TGFβ1 stimulation resulted in a decrease in cytosolic YAP and increased the accumulation of YAP in the nuclear fraction during corneal keratocyte-to-(myo)fibroblast transformation (KFM). A stiffer substrate promoted KFM transformation, which was enhanced in the presence of TGFβ1. Furthermore, YAP can serve as a mechanosensor and mechanotransducer of mechanical cues (Low et al., 2014). In line with these findings, we uncovered that corneal keratocytes easily transform into motile and contractile fibroblasts or myofibroblasts when cultured on stiffer substrates (TCPs) combined with E8 medium. As anticipated, mainly cytoplasmic staining of YAP was observed with a distinct absence of nuclear staining in PDMS-derived spheroids, while localization of YAP was predominant in the nuclei of adherent cells, indicating that, similar to corneal epithelial cells, the phenotype of corneal keratocytes was also dependent upon the mechanical properties of their substrate. The translocation of YAP could be regarded as a hallmark of the stiffness of the ECM. Clinically, in terms of corneal wound healing, the increase in corneal stromal stiffness plays a significant role in promoting KFM transformation and concomitant stromal haze. Raghunathan et al. (2017) inferred that the differentiation of quiescent keratocytes to contractile myofibroblasts might be mediated by YAP/TAZ during wound healing. Our findings suggest that YAP may be a potential treatment target for corneal stromal wound repair. However, it is possible that YAP can convert a range of differentiated cells into somatic stem cells of the same tissue type and also reprogram normal cells into stem-like cells (Panciera et al., 2016; Totaro et al., 2019). Future work will determine whether our current findings are related to YAP expression.

## Conclusion

Our results demonstrated that keratocytes are more susceptible to physical reprogramming than corneal fibroblasts, which may be associated with a transcriptional state favorable to reprogramming. The keratocyte-derived spheroids on PDMS had increased expression of several stemness genes compared to suspended spheroids on ULAP or adherent cells on TCPs. These results indicated that the physiological cells and culture conditions herein enhance reprogramming. We hypothesized that the mechanisms of mechanotransduction, MET and hypoxia induced by PDMS might be responsible for facilitating reprogramming of adult corneal keratocytes. Meanwhile, mechanotransduction induced by PDMS regulates reprogramming enhancement in adherent 3D keratocyte cultures partly through increased histone H3 acetylation. Furthermore, the translocation of YAP between spheroid cells and adherent cells indicates that YAP might be a hallmark of the stiffness of the ECM. Our study provides a biophysical approach without the application of any exogenous genes, RNAs or proteins, which may elucidate how to more safely achieve cellular reprogramming. In the future, we will explore the role of PDMS stiffness and elasticity in cell reprogramming.

## Data Availability

The raw data supporting the conclusion of this article will be made available by the authors, without undue reservation.
