# Supplementary material for: Mechanotransduction Regulates Reprogramming Enhancement in Adherent 3D Keratocyte Cultures
Source: Front Bioeng Biotechnol. 2021 Sep 10;9:709488. doi: 10.3389/fbioe.2021.709488 (PMC8460903; doi:10.3389/fbioe.2021.709488)
Supplement: Supplementary file 1 [file Table1.DOCX]

Table. S1 List of primers

| **Gene** | **Forward Primer（5’—3’）** | **Reverse Primer（5’—3’）** |
| --- | --- | --- |
| *OCT4* | aatttgttcctgcagtgccc | gcagcctcaaaatcctctcg |
| *SOX2* | GCACAACTCGGAGATCAG | CAGCGTGTACTTATCCTTCT |
| *KLF4* | TGAACTGACCAGGCACTA | TCATGTGTAAGGCGAGGT |
| *CMYC* | CACATCAGCACAACTACGCA | GGTGCATTTTCGGTTGTTGC |
| *NANOG* | TCTCCAACATCCTGAACCT | GCGTCACACCATTGCTAT |
| *HNK1* | cctcaactacacgcacctg | ctccaggctgtaggtgttgt |
| *ABCG2* | TGCAACATGTACTGGCGAAGA | TCTTCCACAAGCCCCAGG |
| *PAX6* | CAATCAAAACGTGTCCAACG | TAGCCAGGTTGCGAAGAACT |
| *ALDH3A1* | TGTTCTCCAGCAACGACAAGG | AGGGCAGAGAGTGCAAGGT |
| *KERA* | AACCTGACCCTTCTTGACCT | ACTGCATTGTATTGGCTGGT |
| *CD34* | CTACAACACCTAGTACCCTTGGA | GGTGAACACTGTGCTGATTACA |
| *ALDH1A2* | Tgcttcagaaaggggacgtc | agcccagcctgcgtaatatc |
| *BMP7* | TCGGCACCCATGTTCATGC | GAGGAAATGGCTATCTTGCAGG |
| *FOXO1* | TCGTCATAATCTGTCCCTACACA | CGGCTTCGGCTCTTAGCAAA |
| *LIF* | Ccagatcaggagccaactgg | cacgactatgcggtacagct |
| *NOTCH1* | ACAGATGCCCAGTGAAGC | CGAGGTCAACACAGACGAG |
| *SOX11* | CGGTCAAGTGCGTGTTTCTG | CACTTTGGCGACGTTGTAGC |
| *VEGFA* | Aggccagcacataggagaga | cgcgagtctgtgtttttgca |
| *TGFB1* | AGTTGTGCGGCAGTGGTTGA | GCCATGAATGGTGGCCAGGT |
| *WNT10B* | GTGAGCGAGACCCCACTATG | CACTCTGTAACCTTGCACTCATC |
| *BCL2* | GTGGCCTTCTTTGAGTTCGG | GGCCGTACAGTTCCACAAAG |
| *ID2* | GCTATACAACATGAACGACTGCT | AATAGTGGGATGCGAGTCCAG |
| *MYH9* | GCCGTACAACAAATACCGCT | AGTGTTCCGCTCCTTCTTGA |
| *HIF1A* | CAGTCGACACAGCCTGGATA | GTCCTGTGGTGACTTGTCCT |
| *SMAD3* | ATAACTTGGACCTGCAGCCA | ACATTGGAGAGCAGCCCTAG |
| *MKI67* | AGACCTCCCCAGAGATGGAC | AGACCTCCCCAGAGATGGAC |
| *PCNA* | AACCTGCAGAGCATGGACTC | TATCCGCGTTATCTTCGGCC |
| *DCN* | CGTGCCCATGAGAATGAGATC | AGCAATGCGGATGTAGGAGA |
| *FMOD* | GAGACCTACGAGCCTTACCC | TTGAGGTTGCGATTGTCACAG |
| *GAPDH* | GGAAGGTGAAGGTCGGAGTC | GATCTCGCTCCTGGAAGATGG |
