# Supplementary figures and images for: Mechanotransduction Regulates Reprogramming Enhancement in Adherent 3D Keratocyte Cultures
Source: Front Bioeng Biotechnol. 2021 Sep 10;9:709488. doi: 10.3389/fbioe.2021.709488 (PMC8460903; doi:10.3389/fbioe.2021.709488)

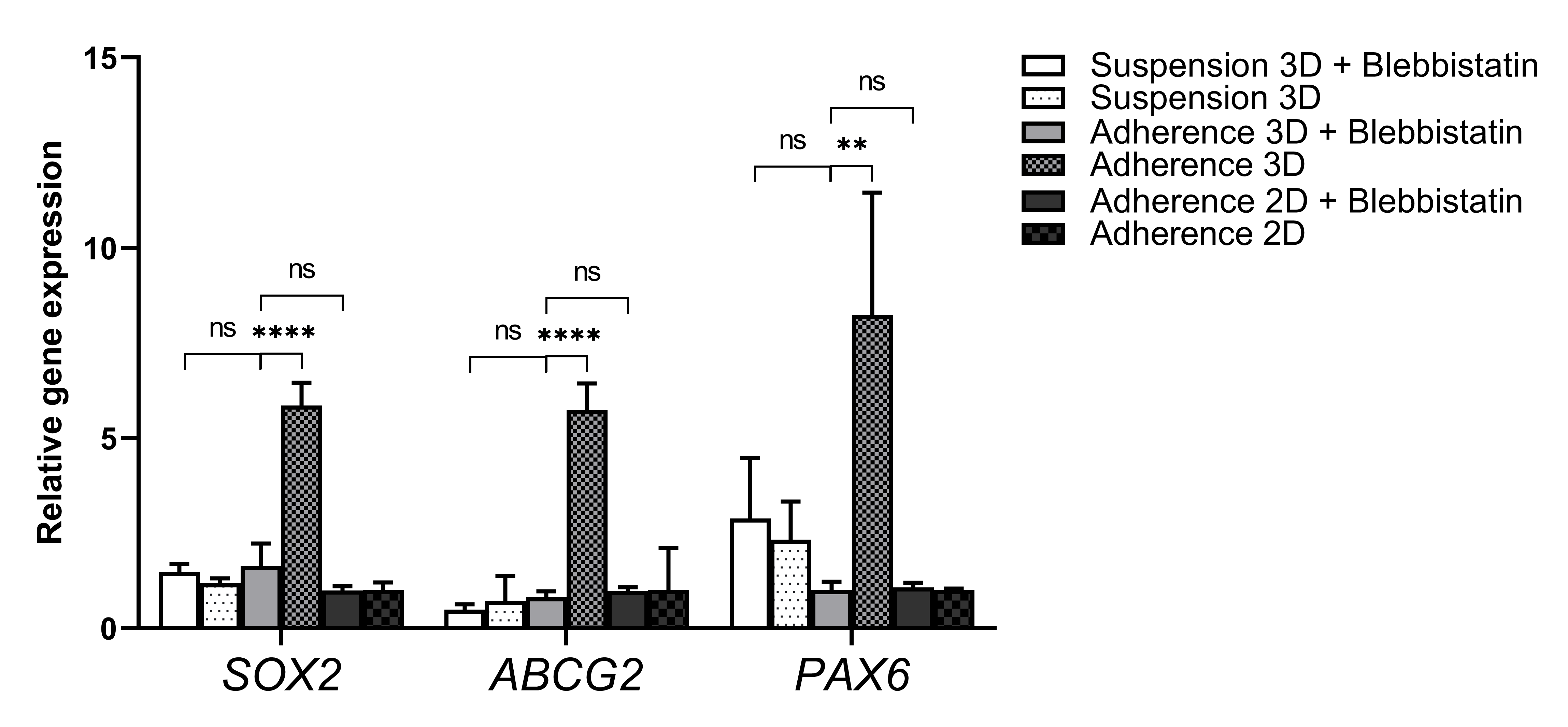

Supplement: Supplementary file 2 [file Image3.TIF]

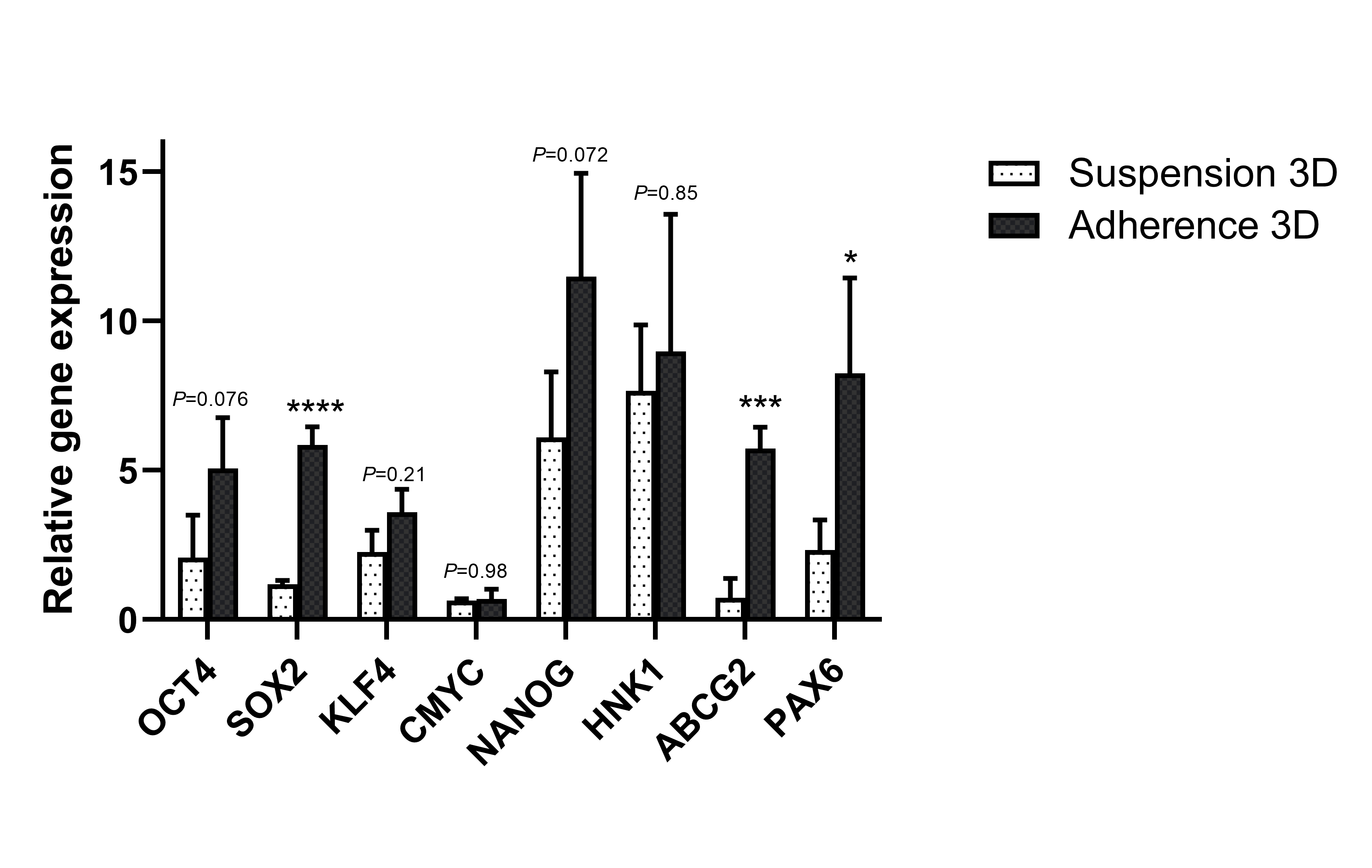

Supplement: Supplementary file 3 [file Image2.TIF]

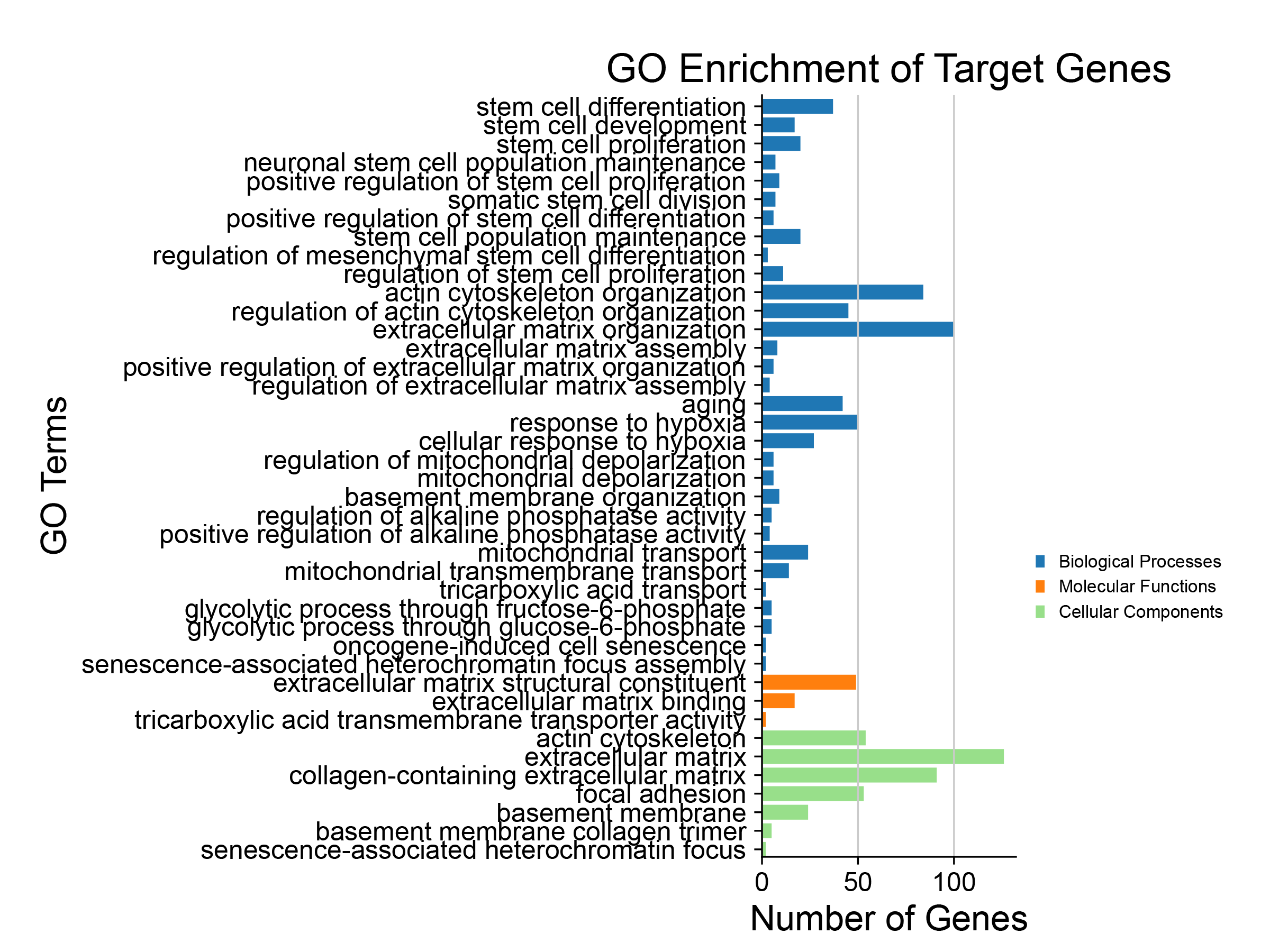

Supplement: Supplementary file 4 [file Image1.TIF]
