# Supplementary material for: Mechanotransduction Regulates Reprogramming Enhancement in Adherent 3D Keratocyte Cultures
Source: Front Bioeng Biotechnol. 2021 Sep 10;9:709488. doi: 10.3389/fbioe.2021.709488 (PMC8460903; doi:10.3389/fbioe.2021.709488)
Supplement: Supplementary file 5 [file DataSheet1.pdf]

Fig. S1 Significant GO term enrichment histograms.

Fig. S2 (A) qPCR analyses showed that *SOX2*, *ABCG2* and *PAX6* were significantly more upregulated in the adherent 3D group than in the suspension 3D group, whereas *OCT4*, *NANOG* and *KLF4* were not significantly different in these two groups. (*, $P < 0.05$; **, $P < 0.01$; ***, $P < 0.001$; ****, $P < 0.0001$).

Fig. S3 qPCR analyses showed the expression of *SOX2*, *ABCG2* and *PAX6* in suspended 3D spheroids, adherent 3D spheroids and adherent 2D cells with or without blebbistatin treatment. (*, $P < 0.05$; **, $P < 0.01$; ***, $P < 0.001$; ****, $P < 0.0001$).
